# Biofortified red mottled beans (*Phaseolus vulgaris *L.) in a maize and bean diet provide more bioavailable iron than standard red mottled beans: Studies in poultry (*Gallus gallus*) and an in vitro digestion/Caco-2 model

**DOI:** 10.1186/1475-2891-10-113

**Published:** 2011-10-14

**Authors:** Elad Tako, Matthew W Blair, Raymond P Glahn

**Affiliations:** 1 USDA/ARS, Robert W. Holley Center for Agriculture and Health, Cornell University, Ithaca, NY 14853, USA; 2 Centro Internacional de Agricultura Tropical (CIAT), Cali, Colombia and Department of Plant Breeding, Cornell University, Ithaca NY 14853, USA

**Keywords:** Beans, biofortification, iron bioavailability, in vitro digestion/Caco- 2 cell model, broiler chicken, intestine

## Abstract

**Background:**

Our objective was to compare the capacities of biofortified and standard colored beans to deliver iron (Fe) for hemoglobin synthesis. Two isolines of large-seeded, red mottled Andean beans (*Phaseolus vulgaris *L.), one standard ("Low Fe") and the other biofortified ("High Fe") in Fe (49 and 71 μg Fe/g, respectively) were used. This commercial class of red mottled beans is the preferred varietal type for most of the Caribbean and Eastern and Southern Africa where almost three quarters of a million hectares are grown. Therefore it is important to know the affect of biofortification of these beans on diets that simulate human feeding studies.

**Methods:**

Maize-based diets containing the beans were formulated to meet the nutrient requirements for broiler except for Fe (Fe concentrations in the 2 diets were 42.9 ± 1.2 and 54.6 ± 0.9 mg/kg). One day old chicks (*Gallus gallus*) were allocated to the experimental diets (n = 12). For 4 wk, hemoglobin, feed-consumption and body-weights were measured.

**Results:**

Hemoglobin maintenance efficiencies (HME) (means ± SEM) were different between groups on days 14 and 21 of the experiment (P < 0.05). Final total body hemoglobin Fe contents were different between the standard (12.58 ± 1.0 mg {0.228 ± 0.01 μmol}) and high Fe (15.04 ± 0.65 mg {0.273 ± 0.01 μmol}) bean groups (P < 0.05). At the end of the experiment, tissue samples were collected from the intestinal duodenum and liver for further analyses. Divalent-metal-transporter-1, duodenal-cytochrome-B, and ferroportin expressions were higher and liver ferritin was lower (P < 0.05) in the standard group vs. the biofortified group. *In-vitro *analysis showed lower iron bioavailability in cells exposed to standard ("Low Fe") bean based diet.

**Conclusions:**

We conclude that the *in-vivo *results support the *in-vitro *observations; biofortified colored beans contain more bioavailable-iron than standard colored beans. In addition, biofortified beans seems to be a promising vehicle for increasing intakes of bioavailable Fe in human populations that consume these beans as a dietary staple. This justifies further work on the large-seeded Andean beans which are the staple of a large-region of Africa where iron-deficiency anemia is a primary cause of infant death and poor health status.

## Introduction

Iron (Fe) deficiency is the most common nutrient deficiency worldwide [1]. A major cause of Fe deficiency is low bioavailability from plant-based diets containing mineral absorption inhibitors such as polyphenols. Policies aimed to decrease Fe deficiency occurrence comprise primarily in the use of dietary iron additives for at-risk populations, food fortification, and diversification of diets. However, these strategies have met with limited success in resource mostly in poor countries because of cost, limited access to health care, partial availability of centralized food processing facilities required for post-harvest crop fortification, and other factors [2-4]. Biofortification, or the crop improvement and breeding of iron-rich staples, is an attractive alternative to fortification or diversification of the diet, since delivery of the iron-rich staple is achieved through variety release and seed promotion.

The common bean (*Phaseolus vulgaris *L*)*, provides significant quantities of protein and energy and is a source of vitamins and minerals including Fe [5]. The common bean is an attractive candidate for Fe biofortification because there is genetic variability of Fe concentration and therefore it is possible to breed for significant increases in Fe concentrations in beans [6]. Also, Fe concentrations in beans are high relative to the cereals and therefore beans can deliver substantial increased amounts of Fe. Bean genotypes with high Fe concentrations delivered more absorbed Fe to rats than genotypes with lower concentrations of Fe [6].

Recently, breeding at CIAT (Centro Internacional de Agricultural Tropical, Cali, Columbia) has developed biofortified beans that contain up to 100 μg Fe/g bean, a substantial increase over standard beans [7,8]. However, an increase in Fe concentration in beans or other staple food crops may not necessarily translate into a proportional increase in absorbed Fe because genotypes with high Fe concentrations may also have increased (or decreased) concentrations of Fe absorption inhibitors or enhancers. Therefore, it is necessary to measure the amount of bioavailable Fe as well as the concentration of Fe in these new iron-enhanced crops.

A previous study aimed to compare the capacities of biofortified and standard small-seeded black beans to deliver Fe for hemoglobin synthesis in Fe deficient pigs, indicated that the pigs receiving the high-Fe bean diet gained significantly more hemoglobin Fe than the piglets on the diet containing standard beans. This result demonstrates that Fe biofortified beans can enhance Fe status in Fe- deficient pigs even when fed as part of a complete diet where the difference in Fe concentration between the diets was only 12 μg/g and the feeding period was only 5 weeks [7]. Similar results using *in vivo *and *in vitro *systems would also be pertinent analytical tools for testing biofortified varieties and the results of Caco-2 cell testing has already been applied to a set of common bean varieties showing significant differences in bioavailability [9]. The objective of this study was to compare the capacities of biofortified and standard, large-seeded, red- mottled bean lines to deliver Fe for hemoglobin synthesis and to improve the Fe status of Fe deficient broiler chickens. In addition, we aimed to determine if the *in vitro *observations of bean Fe bioavailability would be evident in an *in vivo *long term feeding trial.

## Materials and methods

### Animals, Diets and Study Design

Thirty six Cornish cross fertile broiler eggs were obtained from a commercial hatchery (Moyer's chicks, Quakertown, PA). The eggs were incubated under optimal conditions at the Cornell University Animal Science poultry farm incubator. Upon hatching (hatchability rate was 92%), chicks were allocated into 2 treatment groups on the basis of body weight, gender and blood hemoglobin concentration (aimed to ensure equal distribution between groups, n = 12): 1. "High-Fe": 60% red-mottled bean diet (54ppm Fe); 2. "Low-Fe": 60% red-mottled bean diet (42ppm Fe). Experimental diets had no supplemental Fe. Diets composition are shown in Table 1.

**Table 1:** Composition of experimental diets

Ingredient	Low-Fe Bean Diet	High-Fe Bean Diet
**g/kg (by formulation)**		

High-Fe Beans (71 μg Fe/g)	_	600

Low-Fe Beans (49 μg Fe/g)	600	_

Corn	200	200

Corn oil	30	30

Dry skim milk	100	100

Vitamin/mineral premix (no Fe) ^1^	70	70

_DL_-Methionine	2.5	2.5

Choline Chloride	0.75	0.75

Total (g)	1000	1000

Concentrations of selected components	mean ± SEM, n = 5 (by analysis)^4^	

Fe (μg Fe/g) ^2^	42.9 ± 1.2^a^	54.6 ± 0.9^b^

Total Phenols (gallic acid, μg/g) ^3^	103.5 ± 5.5^a^	101.8 ± 6.1^a^

Phytate:Fe molar ratio ^3^	8.28 ± 9.2^a^	8.59 ± 1.06^a^

^1^Vitamin and mineral premix provided/kg diet (330002 Chick vitamin mixture; 230000 Salt mix for chick diet; *Dyets *Inc. Bethlehem, PA).

^2^Iron concentrations in the diets were determined by an inductively-coupled argon-plasma/atomic emission spectrophotometer (ICAP 61E Thermal Jarrell Ash Trace Analyzer, Jarrell Ash Co. Franklin, MA) following wet ashing.

^3^Method for determining phenol concentrations and phytate contents are described in the materials and methods section.

^4^Within a row, means without a common letter are significantly different, P < 0.05.

Chicks were housed in a total-confinement building (1 chick per 0.5 m^2 ^metal cage). Birds were under indoor controlled temperatures and were provided 16 h of light. Each cage was equipped with an automatic nipple drinker and manual self feeder. All birds were given *ad libitum *access to water (Fe content was 0.379 ± 0.012ppm). Feed intakes were measured daily (as from day 1). Iron intakes were calculated from feed intakes and Fe concentration in the diets.

The two red-mottled bean lines used in the study were developed from a commercial variety for Africa (CAL96, Low-Fe that has been released in Uganda and widely used in crosses by CIAT, ECABREN and SABRN) and an introgressed line (NUA35, High-Fe) from the backcross CAL96 × (CAL96 × G14519) as described in [8]. Seed was multiplied in Darien, Colombia under phosphorus fertilized, standard agronomic conditions and shipped to Ithaca, New York in sealed containers imported as grain.

### Blood Analysis and Hemoglobin (Hb) Measurements

Blood samples were collected weekly from the wing vein (n = 12, ~100 μL) using micro-hematocrit heparinized capillary tubes (Fisher, Pittsburgh, PA). Samples were collected in the morning following an 8 h overnight fast. The samples were analyzed for hemoglobin (Hb) concentration (see below). Body weights and hemoglobin concentrations were measured weekly.

Fe bioavailability was calculated as hemoglobin maintenance efficiency (HME) [7,10-14]:

$$HME=\frac{Hb\;Fe,\;mg\;\left(final\right)-Hb\;Fe,\;mg\;\left(initial\right)}{Total\;Fe\;Intake,\;mg}\times 100$$

Where Hb-Fe (index of Fe absorption) = total body hemoglobin Fe. Hb-Fe was calculated from hemoglobin concentrations and estimates of blood volume based on body weight (a blood volume of 85 mL per kg body weight is assumed) [12-14]:

$$\text{Hb}-\text{Fe}\left(\text{mg}\right)=\text{B}.\text{W}.\left(\text{kg}\right)\times 0.0\text{85 L blood}∕\text{kg}\times \text{Hb}\left(\text{g}∕\text{L}\right)\times \text{3}.\text{35mg Fe}∕\text{g Hb}.$$

Fe intakes were calculated from feed intake data and Fe concentrations in the feed.

At the end of the experiment (day 28), birds were euthanized by carbon dioxide exposure. The digestive tracts and livers were quickly removed from the carcass and separated into various sections for tissue (small intestine and liver ~ 1-2 cm; ~2-3gr, respectively). The samples were immediately frozen in liquid nitrogen, and then stored in a -80°C freezer until analysis.

All animal protocols were approved by the Cornell University Institutional Animal Care and Use Committee.

Blood Hb concentrations were determined spectrophotometrically using the cyanmethemoglobin method (H7506-STD, Pointe Scientific Inc. Canton, MI) following the kit manufacturer's instructions.

### Isolation of Total RNA

Total RNA was extracted from 30 mg of the duodenal tissue (tissue was harvested from the proximal duodenum, n = 6) using Qiagen RNeasy Mini Kit (RNeasy Mini Kit, Qiagen Inc., Valencia, CA)^1 ^according to the manufacturer's protocol. Briefly, tissues were disrupted and homogenized with a rotor-stator homogenizer in buffer RLT^®^, containing β-mercaptoethanol. The tissue lysate was centrifuged for 3 minutes at 8,000 g in a micro centrifuge. An aliquot of the supernatant was transferred to another tube, combined with 1 volume of 70% ethanol and mixed immediately. Each sample (700 μL) was applied to an RNeasy mini column, centrifuged for 15 s at 8000 g, and the flow through material was discarded. Next, the RN easy columns were transferred to new 2-mL collection tubes, and 500 μL of buffer RPE^® ^was pipetted onto the RNeasy column followed by centrifugation for 15 s at 8000 g. An additional 500 μL of buffer RPE were pipetted onto the RNeasy column and centrifuged for 2 min at 8000 g.

Total RNA was eluted in 50 μL of RNase free water. All steps were carried out under RNase free conditions. RNA was quantified by absorbance at *A _260/280_*. Integrity of the 28S and 18S ribosomal RNAs was verified by 1.5% agarose gel electrophoresis followed by ethidium bromide staining. DNA contamination was removed using TURBO DNase treatment and removal kit from AMBION (Austin, TX, USA).

### DMT1, DcytB and Ferroprtin Gene Expression Analysis

As previously described [12,14,15], briefly, PCR was carried out with primers chosen from the fragment of the chicken (*Gallus gallus) *duodenal DMT1 gene (GeneBank database; GI 206597489) (forward: 5'-AGC CGT TCA CCA CTT ATT TCG-3'; reverse: 5'-GGT CCA AAT AGG CGA TGC TC-3'), DcytB gene (GI 20380692) (forward: 5'-GGC CGT GTT TGA GAA CCA CAA TGT T-3'; reverse: 5'-CGT TTG CAA TCA CGT TTC CAA AGA T-3') and Ferroportin gene (GI 61098365) (forward: 5'-GAT GCA TTC TGA ACA ACC AAG GA'; reverse: 5'-GGA GAC TGG GTG GAC AAG AAC TC-3'). Ribosomal 18S was used to normalize the results (GI 7262899) (forward: 5'- CGA TGC TCT TAA CTG AGT-3'; reverse: 5'-CAG CTT TGC AAC CAT ACT C-3').

Determination of the linear phase of the PCR amplification was performed (Access RT-PCR system, Promega, Madison, WI) with pooled aliquots removed at 15, 20, 25, 30, 35, 40, 45, 50, and 55 cycles. Amplification of the chicken duodenal DMT1, DcytB and Ferroportin genes were performed for 32, 40 and 30 cycles respectively, which consisted of denaturation (95°C, 30 s), annealing (48°C, 1 min), and extension (72°C, 1 min); ribosomal 18S was amplified at 32 cycles under identical conditions in a different tube. All PCR products were separated by electrophoresis on 2% agarose gel, stained with ethidium bromide, and quantified using the Quantity One 1-D analysis software (Bio-Rad, Hercules, CA).

### In-vitro Iron Bioavailability Assessment

An in vitro digestion/Caco-2 cell culture model [16] was used to assess *in vitro *iron bioavailability. With this method, the cooked bean samples of the two diets were subjected to simulated gastric and intestinal digestion. Briefly, the intestinal digestion is carried out in cylindrical inserts closed on the bottom by a semipermeable membrane and placed in wells containing Caco-2 cell monolayers bathed in culture medium. The upper chamber was formed by fitting the bottom of Transwell insert ring (Corning) with a 15000 Da molecular weight cut off (MWCO) membrane (Spectra/Por 2.1, Spectrum Medical, Gardena, CA). The dialysis membrane was held in place using a silicone ring (Web Seal, Rochester, NY).

Iron uptake by the cell monolayers was assessed by measuring ferritin concentrations in the cells. Six replicates of each Fe bioavailability measurement were performed. In terms of materials for the study, Caco-2 cells were obtained from the American Type Culture Collection (Rockville, MD) at passage 17 and used in experiments at passage 29. Cells were seeded at densities of 50,000 cells/cm^2 ^in collagen-treated 6 well plates (Costar Corp., Cambridge, MA). The integrity of the monolayer was verified by optical microscopy. The cells were cultured at 37°C in an incubator with 5% CO_2 _and 95% air atmosphere at constant humidity, and the medium was changed every 48 h.

The cells were maintained in Dulbecco's modified Eagle medium plus 1% antibiotic/antimycotic solution, 25 mmol/L HEPES, and 10% fetal bovine serum. 48 h prior the experiment, the growth medium was removed from culture wells, the cell layer was washed, and the growth medium was replaced with minimum essential media (MEM) at pH 7.0. The MEM was supplemented with 10 mmol/L PIPES, 1% antibiotic/antimycotic solution, 4 mg/L hydrocortisone, 5 mg/L insulin, 5 μg/L selenium, 34 μg/L triiodothyronine, and 20 μg/L epidermal growth factor. This enriched MEM contained less than 80 μg Fe/L.

All ingredients and supplements for cell culture media were obtained from Gibco (Rockville, MD). The cells were used in the Fe uptake experiment at 13 days post seeding. In these conditions, the amount of cell protein measured in each well was highly consistent between wells. In experiment day, 1.5 mL of the digested sample was added to the insert's upper chamber and incubated for 2h. Then, inserts were removed and 1 mL of MEM was added. Cell cultures were incubated for 22h at 37°C.

It was previously shown that intracellular ascorbic acid status might influence ferritin formation (i.e. cellular Fe uptake), and Fe related transporters and enzyme expression in Caco-2 cells [17,18]. In the current study, samples were not added with ascorbic acid when Fe bioavailability was tested *in vitro*.

### Harvesting of Caco-2 Cells for Ferritin Analysis

The protocols used in the ferritin and total protein contents analyses of Caco-2 cells were similar to those previously described [14,19]. Briefly, growth medium was first removed from the culture well by aspiration and the cells were washed twice with a solution containing 140 mmol/L NaCl, 5 mmol/L KCl, and 10 mmol/L PIPES at pH 7.0. The cells were harvested by adding an aliquot of deionized water and placing them in a sonicator (Lab-Line instruments, Melrose Park, IL).

The ferritin and total protein concentrations were determined on an aliquot of the harvested cell suspension with a one-stage sandwich immunoradiometric assay (FER-IRON II Ferritin assay, Ramco laboratories, Houston, TX) and a colorimetric assay (Bio-Rad DC Protein assay, Bio-Rad, Hercules, CA), respectively. Caco-2 cells synthesize ferritin in response to increases in intracellular iron concentration. Therefore, we used the ratio of ferritin/total protein (expressed as ng ferritin/mg protein) as an index of the cellular Fe uptake. All glassware used in the sample preparation and analyses was acid washed.

### Ferritin and Fe in the Liver

Liver samples were treated as described by Mete et al. [20]. Briefly, 1 g of sample was diluted into 1 mL of 50 mM Hepes buffer, pH 7.4, and homogenized on ice at 5000 g and for 2 min. One mL of each homogenate was subjected to heat treatment for 10min at 75°C to aid isolation of ferritin since other proteins are not stable at that temperature [14,15,20,21]. After heat treatment the samples were immediately cooled down on ice for 30min. Thereafter, samples were centrifuged at 13000 g for 30min at 4°C until a clear supernatant was obtained and the pellet containing most of the insoluble denaturated proteins was discarded. All tests were conducted in duplicates for each animal (n = 6).

### Electrophoresis, Staining and Measurement of Gels

Native polyacrylamide gel electrophoresis was conducted using a 6% separating gel and a 5% stacking gel. Samples were run at a constant voltage of 100 V. After electrophoresis, the gels were treated with either of the two stains: Coomasie blue G-250 stain, specific for proteins, or potassium ferricyanide (K_3_Fe(CN)_6_) stain, specific for iron. The corresponding band found in the protein and iron stained gel was considered to be ferritin [20,21].

The gels were scanned with Bio-Rad densitometer. Measurements of the bands were conducted using the Quantity-One 1-D analysis program (Bio-Rad, Hercules, CA). The local background was subtracted from each sample. Horse spleen ferritin (Sigma Aldrich Co., St. Louis, MO) was used as a standard for calibrating ferritin protein and iron concentrations of the samples. Dilutions of the horse spleen ferritin were made and treated similarly to the liver supernatant samples in order to create a reference line for both protein and iron-stained gels. Iron levels were determined using the same calibration since approximately 20% of the weight of horse spleen ferritin is iron [21,22]. Saturation levels of ferritin with iron were calculated as the percentage of the iron present in the protein to the maximum amount of iron atoms (4500 iron atoms/ferritin molecule) ferritin can incorporate [14,15,20].

### Polyphenol Concentrations in Diets

Samples (n = 5, 1.5 g) of the diets were extracted with 4 mL of acidified methanol (methanol and 1.0 M HCl, 85:15 v/v). The samples were shaken for 2h, vortexed to mix, and centrifuged at 12800 g for 10 min to remove insoluble material.

To evaluate the total phenols content in the extracts, we used the following method [10,14,22]; Briefly, to 0.125 mL of the supernatant, 0.5 mL of deionized water and 0.125 mL of the Folin-Ciocalteau reagent were added. The mixture was left at room temperature for 5min, and then, 0.125 mL of aqueous Na_2_CO_3 _(7% wt/vol) was added. The final volume of the mixture was adjusted to 3 mL with deionized water and was left at room temperature for 1.5h. The absorbance was measured at 760 nm against a reagent blank. The amount of total phenolics was expressed as gallic acid equivalents (μg/g of sample).

### Phytate Content in Diets

A Dionex liquid (Dionex Corp. Sunnyvale, CA) chromatograph system (AS50 autosampler), equipped with conductivity detector model ED50, and gradient pump GS50 were used along with an IonPac AG11 guard column and IonPac AS11 column (4 × 250 mm) to quantify phytate. PeakNet 6.40 software was used to process chromatographic data. The mobile phases were (A) 200 mmol/L NaOH (carbonate-free) and (B) deionized water, using a flow rate of 1 mL/min. Phytate was extracted from 250 mg of dry, lyophilized diet sample, in 10 mL of a 1.25% H_2_SO4 solution; the extraction process was 2h, after which the samples were centrifuged at 3660*g *for 10 min. Subsamples were diluted 1:10 with deionized water, and 10*μ*L was injected and analyzed (n = 5).

### Statistical Analysis

Results were analyzed by ANOVA using the general linear models procedure of SAS software (SAS Institute Inc. Cary, NC). Differences between treatments were compared by Tukey's test and values were considered statistically different at P < 0.050 (values in the text are means ± SEM).

## Results

### Growth rates, hemoglobin (Hb), Hb Fe and Hb maintenance efficiency (HME)

There were no significant differences in feed intakes at any time throughout the study; however, Fe intakes were consistently higher in the "High-Fe" group vs. "Low-Fe" group (Table 2). Hemoglobin concentrations were higher (P > 0.05) in the "High-Fe" group vs. "Low-Fe" group. Significant differences were measured in HME on days 14 and 21 of the experiment between the "High-Fe" group vs. "Low-Fe" treatment (Table 2).

**Table 2:** Body weights, Fe intakes, hemoglobin maintenance efficiency (HME), and total body Hb Fe content in chicken fed the tested diets from d 0 to d 56^1^.

Treat^*^	Day 0	Day 7	Day 14	Day 21	Day 28
Body weight (g)					

High Fe	42.5^a ^± 2.1	107.6^a ^± 3.9	214.6^a ^± 5.8	427.8^a ^± 16.8	684.3^a ^± 21.9

Low Fe	42.9^a ^± 2.6	103.9^a ^± 4.3	204.1^a ^± 9.1	398.7^a ^± 18.8	599.9^b ^± 26.1

Feed intake (kg/d)^2^					

High Fe	-	0.02^a ^± 0.005	0.07^a ^± 0.008	0.07^a ^± 0.01	0.08^a ^± 0.01

Low Fe	-	0.02^a ^± 0.006	0.06^a ^± 0.007	0.07^a ^± 0.009	0.08^a ^± 0.01

Fe intake (mg) ^3^					

High Fe	-	9.6^a ^± 0.7	33.8^a ^± 1.5	58.7^a ^± 4.9	87.6^a ^± 6.5

Low Fe	-	7.0^b ^± 0.5	23.3^b ^± 1.8	44.4^b ^± 3.8	65.6^b ^± 4.9

Hemoglobin (g/L)					

High Fe	90.50^a ^± 8.3	85.85^a ^± 4.0	79.60^a ^± 4.5	80.14^a ^± 6.5	75.50^a ^± 3.1

Low Fe	90.70^a ^± 8.7	78.40^a ^± 4.7	76.10^a ^± 3.7	78.40^a ^± 2.3	73.71^a ^± 2.3

Total body Hb Fe content (mg)^4^					

High Fe	1.08^a ^± 0.06	2.62^a ^± 0.18	4.83^a ^± 0.31	9.77^a ^± 0.58	15.04^a ^± 0.65

Low Fe	1.11^a ^± 0.08	2.36^a ^± 0.24	4.58^a ^± 0.29	8.50^b ^± 0.35	12.58^b ^± 1.03

Hemoglobin maintenance efficiency (HME, %)^5^					

High Fe	-	16.0^a ^± 1.9	11.0^a ^± 0.9	14.7^a ^± 1.0	15.9^a ^± 0.7

Low Fe	-	18.5^a ^± 3.8	15.1^b ^± 1.2	16.7^b ^± 0.7	17.6^a ^± 1.3

^a, b ^Within a column, means without a common letter are significantly different, P < 0.05.

^1^Values are means ± SEM, n = 12.

^2^Values are mean daily feed intakes for the 7 days proceeding the day designated in the column heading.

^3^Values are mean ± SEM (cumulative weekly from day 0)

^4,5^Fe bioavailability was calculated as hemoglobin maintenance efficiency (*HME*) (7,10-14):

$$\text{HME}=\frac{Hb\;Fe,\;mg\;\left(final\right)-Hb\;Fe,\;mg\;\left(initial\right)}{Total\;Fe\;Intake,\;mg}\times 100$$

Where Hb-Fe = total body hemoglobin Fe. Hb Fe was calculated from hemoglobin concentrations and estimates of blood volume based on body weight (a blood volume of 85 mL per kg body weight is assumed) (10,11,14,23):

$$\text{Hb}-\text{Fe}\left(\text{mg}\right)=\text{B}.\text{W}.\left(\text{kg}\right)\times 0.0\text{85 L blood}∕\text{kg}\times \text{Hb}\left(\text{g}∕\text{L}\right)\times \text{3}.\text{35mg Fe}∕\text{g Hb}.$$

* The experimental diets: 1. "High Fe": 60% colored bean diet (54ppm Fe); 2. "Low Fe": 60% colored bean diet (42ppm Fe).

In addition, the increase in total body Hb Fe from the beginning of the study to the end of the 4th week was significantly greater in the "High-Fe" group vs. "Low-Fe" group (12.6 ± 0.7 mg and 10.2 ± 0.4 mg, respectively, P < 0.05, Table 2).

### Gene expression of iron transporters (DMT-1, Ferroportin) and DcytB in the duodenum

**Figure 1:**
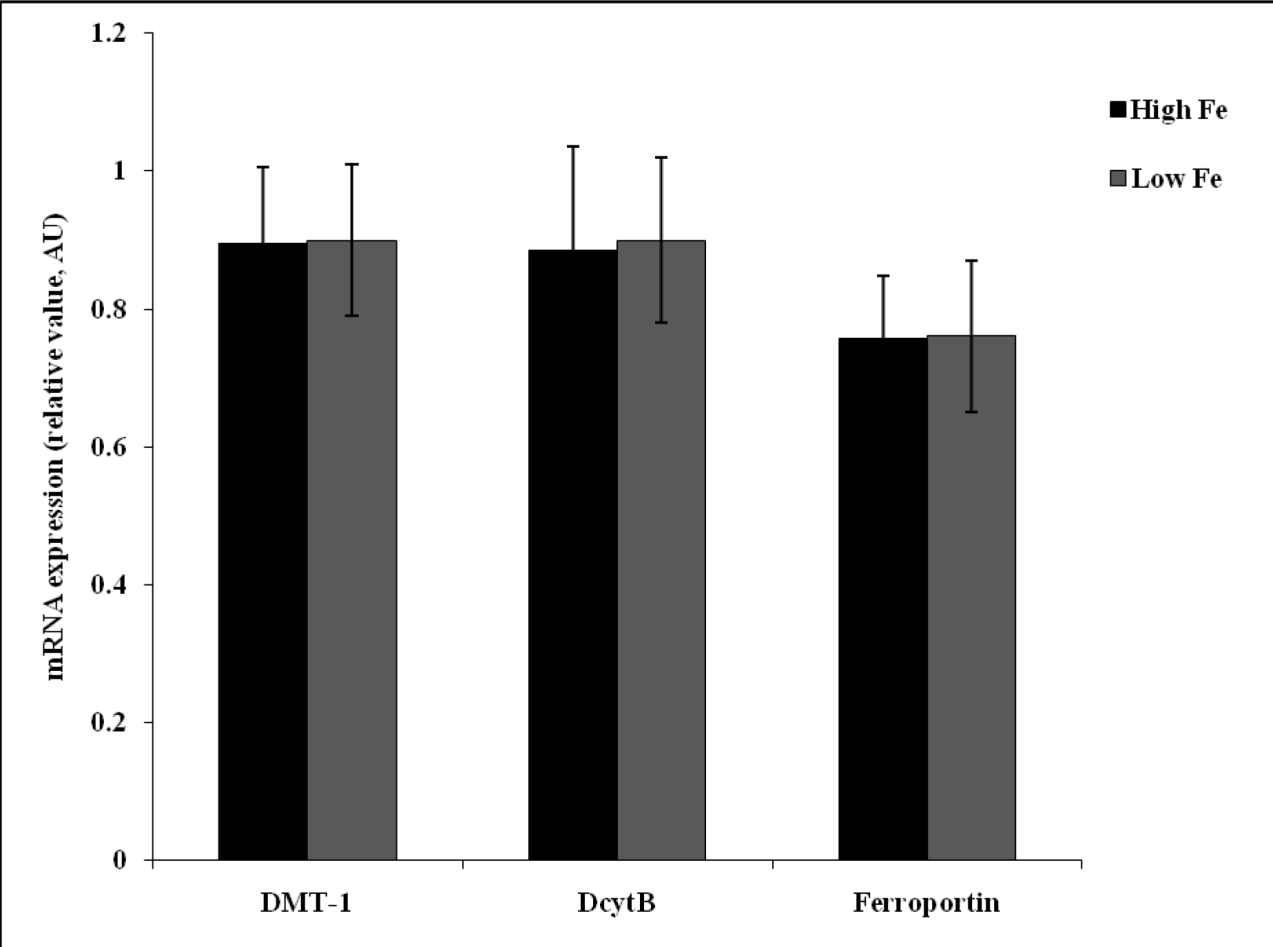
**Chicken duodenal mRNA expression of divalent metal transporter 1 (DMT1), ferroportin, and duodenal cytochrome B (DcytB) in birds given "High Fe bean" diet (54.6 ppm Fe) and "Low Fe bean" diet (42.9 ppm Fe)**. Changes in mRNA expression are shown relative to expression of 18S rRNA in arbitrary units (AU). Values are means ± SEM, n = 6, P < 0.05.

Gene expression analysis of duodenal DMT-1, Ferroportin and DcytB, with results reported relative to 18S rRNA, revealed increased mRNA expression of DMT1, DcytB and Ferroportin in the "Low-Fe" group compared to "High Fe" group (Figure 1) (n = 6, P > 0.05).

### Caco-2 cell ferritin protein formation

An *in vitro *digestion/Caco-2 cell culture model was used to evaluate Fe bioavailability from the test diets by measuring ferritin formation in the cells (ie. a measure of cell Fe uptake) following exposure to digests of the samples. Ferritin concentrations were significantly higher (P < 0.05) in cells exposed to the "High Fe" group vs. "Low Fe" group (n = 6, Table 3).

**Table 3:** Ferritin concentrations in Caco 2 cells exposed to samples of beans only (whole bean, 49 ppm Fe and 71 ppm Fe, "Low Fe" and "High Fe" beans, respectively) and bean-based diets digests; "Low Fe" bean diet (42 ppm Fe) and "High Fe" bean diet (54 ppm Fe).

**Tested sample**^**1**^	Ferritin (ng/mg of protein)
"Low Fe" bean only	7.82 ± 0.75^d^

"High Fe" bean only	30.6 ± 2.08^a^

"Low Fe" bean-based diet	11.2 ± 0.97^c^

"High Fe" bean-based diet	15.7 ± 1.05^b^

Baseline^2^	4.06 ± 0.37^e^

^1^Caco 2 bioassay procedures and preparation of the digested samples are described in the materials and methods sections.

^2^Cells were exposed to only MEM growth media without added food digests and Fe. Values are means ± SEM, n = 6. Means with no letters in common are significantly different, P < 0.05.

### Ferritin and iron in the liver

The avian ferritins corresponded to a weight of approximately 470 to 500 kDa [14,15,20-22]. No significant differences in liver Fe or liver ferritin concentrations were measured between the treatment groups (n = 6, P > 0.05). The mean values of ferritin protein and the amount of iron present in the ferritin of the liver samples of all animals are presented in Table 4.

**Table 4:** Ferritin protein and the iron^1 ^concentration in the liver tissue in birds given "High Fe" diet (54 ppm Fe) and "Low Fe" (42 ppm Fe)(values are mean ± SEM, n = 6)

Treatment diet	Ferritin μg/g wet weight	Iron μg/g wet weight	Iron/Ferritin μmol
"High Fe"	425 ± 18^a^	48.1 ± 4.2^a^	68.5 ± 5.6^a^

"Low Fe"	409 ± 12^a^	39.5 ± 3.5^a^	59.8 ± 5.2^a^

^1^Atomic mass for iron is 55.8 g/mol.

### Total phenolic concentration in the diet sample

The total phenolics in the bean-based diet samples are expressed as gallic acid equivalents (μg/g of sample, mean ± STD, n = 5) and were as follows: 103.5 ± 5.5 μg/g diet and 101.8 ± 6.1 μg/g diet in the " Low Fe", and "High Fe", respectively (P < 0.05) as shown in Table 1.

### Phytate concentration in the diet samples

No significant differences in phytate concentration were measured between treatments diets (n = 5; P > 0.05). Dietary phytate concentrations (IP_6_) are shown in Table 1.

## Discussion

The main objective of the present study was to compare the capacities of biofortified ("High Fe") and standard ("Low Fe") large-seeded, red-mottled beans that differ in Fe concentration (71 μg/g vs. 49 μg/g, respectively) to deliver Fe for Hb synthesis and to improve the Fe status of Fe-deficient broiler (*Gallus gallus*) chickens. These beans are an important commercial class for human consumption in Eastern and Southern Africa. In addition, they are grown in the Caribbean and the Andean region of South America where they originated as a specific group of common bean [8].

Our data showed that Fe deficient birds receiving the high Fe bean diet gained significantly more Hb Fe than the birds on the diet containing standard beans. This result clearly shows that Fe biofortified red-mottled beans can enhance Fe status in Fe-deficient birds even when fed as part of a complete diet, where the difference in Fe concentration between the diets was only 12 μg/g and the feeding period was only 4 weeks.

In addition, it was previously shown that colored beans are rich with polyphenols that may decrease Fe bioavailability both *in vitro *[9] or *in vivo *[14]. According to the present study, it appears that it is possible to counteract the Fe absorption inhibitory effect of polyphenols by increasing Fe concentration in beans. This knowledge is vital for developing plant breeding strategies as part of the continuing battle with dietary Fe deficiency anemia.

Furthermore, the results obtained in the present study are consistent with a previous study where we have demonstrated that Fe biofortified black beans delivered more bioavailable Fe than standard black beans to Fe deficient piglets and improved their Fe status by increasing the piglets total body Hb Fe content. Similar to the current study, the difference in Fe concentration between the two diets (standard vs. biofortified black beans) was 12 μg/g, however, the duration of the study was 5 weeks [7]. Importantly, the previous work was performed with small-seeded beans which have a higher ratio of seed coat to total seed weight than the large-seeded beans used in this study which may affect bioavailability due to a greater percentage of polyphenols per seed weight [9].

In a different study, Schaffer et al. [23] compared the effects of high Fe (13.4 μg/g) and low Fe (2.2 μg/g) rice on Fe status indices in weaned piglets. At the end of the 33 days feeding trial, none of the indices differed. A possible explanation for the lack of effect in this study is that both diets were extremely deficient in Fe; Hb concentrations at the end of the study were only 45 g/L. In contrast, in a study conducted by Haas et al. [24] Fe-biofortified rice improved Fe stores in Fe-deficient but not anemic Filipino women in a nine months efficacy trial, even though Fe concentrations in the polished rice they used were extremely low (3.2 μg/g for the high Fe rice and 0.57 μg/g for the control rice).

The longer feeding duration in the Haas study [24] and lower Fe requirements in adult women compared with early weaned pigs may explain why they found an effect whereas Schaffer et al. [23] did not.

In the current study and in order to demonstrate a possible nutritional benefit of the biofortified red-mottled beans (i.e. improving Fe status), we first tested the Fe bioavailability by measuring cellular ferritin concentrations in Caco-2 cells exposed to the red-mottled beans and high *vs*. low bean-based diets. This *in-vitro *method has been used to screen food crops as part of the plant breeding strategy aimed to alleviate micronutrient deficiencies in relevant populations [7,9-12,14,16,19]. Based on the *in-vitro *observations showing higher ferritin concentrations in cells exposed to the high Fe bean and high Fe bean based diet (Table 3), we designed an *in-vivo *study aimed to determine if the *in-vitro *observations of bean Fe bioavailability would be evident in an *in-vivo *feeding trial.

We selected the broiler chicken as a model for Fe bioavailability studies since the modern broiler chicken is a fast growing animal that is sensitive to dietary deficiencies of trace minerals such as Fe [12-15]. As such, it holds potential as a relevant model as a source of tissues for *in-vitro *Fe bioavailability studies, *in-vivo *feeding trials, or both. In addition, the use of broiler chickens for *in-vivo studies *represents a significant cost savings compared to studies with piglets or human models.

Furthermore, we previously demonstrated that the use of the poultry model for Fe bioavailability studies has numerous applications [12-15], but in general it can be used to identify foods, food combinations and factors within diets that can help prevent Fe deficiency anemia. Therefore, it may be especially useful in the strategy of "biofortification". This approach utilizes plant breeding to select for traits that enhance the nutritional quality of crops by increasing Fe concentration and or bioavailability [24] and requires an inexpensive method that can screen more than the two to ten advanced lines proposed for varietal release.

In biofortification studies, the effect of a biofortified food (i.e. nutritional benefit) is expected to be preventative; thus, depending on the duration of the study and as previously suggested, Fe deficient (not anemic) animals are preferred [7,24,25]. Anemic animals are not desirable for biofortification studies because physiological adaptation may mask differences in bioavailable Fe between test samples [10,11]. Also, the difference in deliverable Fe between samples may not be enough to reverse the anemia, or require a longer time to show a measurable benefit. Alternatively, Fe-adequate animals may take a long time to show depletion of Fe, thus less effect would be shown during a study. Therefore, the initial Fe status should be established to accommodate possible changes in Fe status and thus maximize the potential for measurement of physiological effects.

Based on the above, one of the parameters we chose to use as a physiological marker of Fe status was the increase in body Fe in the birds as an index of Fe absorption. To do this, we needed to accurately measure the accumulation of Fe in the animal over an extended feeding period. Our rationale was that if we could keep our birds Fe deficient, then their Fe stores should be minimal and we could use Hb Fe and tissue Fe levels as a reasonable index of absorbed Fe. This approach is based on the concept that storage Fe is almost completely depleted before Fe deficiency anemia develops [25]. We monitored the Fe status of the chicks so that they would be Fe-deficient (and not anemic) at the start of the study; broiler hatchlings grow rapidly and therefore have very high Fe requirements, hence, at hatch broiler chicks are Fe deficient [15] and without the appropriate diet will develop severe Fe deficiency [11].

In this study, the mean Hb concentrations in both groups at the start of the feeding period was 91 g/L. This Hb status may increase or decrease dependent on the given dietary Fe concentrations and bioavailability. The Hb concentrations were maintained at this level throughout the study in the group receiving biofortified beans ("High Fe" group) but fell in the standard ("Low Fe") bean group (Figure 1). This suggested a nutritional benefit from the high Fe nutritionally-enhanced, red-mottled beans.

In addition to the physiological parameters mentioned above, we also measured the effect of the experimental diets on Fe transporters and enzyme expression. It was previously established that Fe absorption is regulated, in part, by the intracellular Fe concentrations in the enterocyte [26]. Iron ions (Fe^2+ ^and Fe^3+^) reach the duodenal brush border membrane then are reduced by DcytB to Fe^2+ ^(unless already in the Fe^2+ ^form), which is then transported into the enterocyte via DMT-1. Other mechanisms for Fe entry into enterocytes are possible and likely but have not been conclusively demonstrated [27]. Within the cell, Fe is either stored as ferritin or trafficked to the basolateral membrane and exported into the circulation. Transport across the basolateral membrane is accomplished by the coordinated action of ferroportin, an Fe transporter, and hephaestin, which oxidizes Fe^2+ ^to Fe^3+^. Iron ions (Fe^3+^) then bind to transferrin for distribution throughout the body via the plasma circulation [28,29].

In the current study, the duodenal relative mRNA abundance of DMT-1, DcytB and ferroportin were higher in the "Low Fe" group vs. the "High Fe" group, however, the up regulation was not significant (P > 0.05). We previously showed that increased expression of intestinal Fe related transporters and enzymes indicated on Fe deficiency [12,14,15]. This pattern of expression was observed in the present study and was described in Fe-deficient rats and *in vitro *[30]. It was also shown that the elevated gene expression for these transporters and enzymes is due to the dietary Fe deficiency conditions and increases cellular Fe uptake and export into the circulation [30]. These observations indicate that the Fe uptake mechanisms in the broiler are responding as expected to dietary Fe [11,14,15]. This also implies that the biofortified red beans improved the Fe status of the Fe deficient birds.

Other major parameters for Fe status are liver ferritin and liver Fe concentrations. Ferritin, as the cellular Fe-binding protein, represents the Fe status of the tissue and reflects on the Fe status of the body [20,21,31]. In the present study, we document quantification of liver ferritin; studying the ferritin protein in its native state has allowed us to calculate the Fe bound to its core [14,15,20,32]. Our results showed that birds fed the "High Fe" diet had higher liver ferritin concentrations (P > 0.05). Although this increase in ferritin concentration was not significant, it is still indicates on higher Fe bioavailability in the "High Fe" diet. Also, liver Fe concentrations were lower (P > 0.05) in the "Low Fe" diet group in comparison to "High Fe" group. These observations verify the *in-vitro *model results, indicating that Fe bioavailability was higher in the "High Fe" bean based diet relative to the "Low Fe" bean based diet.

In summary, the current study suggests that increasing Fe concentrations in large-seeded, red-mottled beans by about 25 μg/g should provide more bioavailable and therefore absorbable Fe. Also, increased Fe concentration seems to limit the polyphenolic inhibitory effect on Fe absorption from colored beans. Hence, use of plant breeding programs strategies of selection for high Fe content may have significant nutritional benefits.

## Conclusions

Based on the data shown here, we conclude that the biofortified beans are a promising vehicle for increasing intakes of bioavailable Fe in human populations where beans are a dietary staple and that colored beans contain relatively high levels of bioavailable Fe. An efficacy trial comparing biofortified and standard red-mottled beans in human populations is needed to confirm the findings reported here.

## Abbreviations

Fe: iron; Hb: hemoglobin; Hb: Fe, hemoglobin iron; HME: hemoglobin maintenance efficiency; CIAT: Centro Internacional de Agricultural Tropical; PCR: polymerase chain reaction; DMT-1: divalent metal transporter 1; DcytB: duodenal cytochrome B; Da: dalton; MEM: minimum essential media.

## Competing interests

The authors declare that they have no competing interests.

## Authors' contributions

ET conceptualized the design of the study and study protocol, as well as performed the samples and data collection, statistical analyses and writing of the manuscript. MB provided the bean lines used in the study. The in vitro analysis was done in collaboration with RG that also assisted in editing the manuscript. All the authors read and approved the final manuscript

## Endnotes

^1 ^Mention of a trademark, proprietary product or vendor does not constitute a guarantee or warranty of the product by the United states Department of Agriculture and does not imply its approval to the exclusion of other products or vendors that may also be suitable.
